# The Water Supply of Small Towns

**Published:** 1896-04-25

**Authors:** 


### THE WATER SUPPLY OF SMALL TOWNS.

As civilisation progresses, primitive or what are called "natural" methods of doing even such common things as supplying rural households with water tend to become more and more distrusted and more and more obsolete. The country, so far, has mostly preferred "country" to "town" methods of water supply. Your genuine countryman is immovably confident that no town-bred engineer can furnish him with water which will for a moment compare in quality with that of the trout stream which passes through his pasture, or of the crystal spring that gushes out fresh and sparkling all the year round from the natural rockery at the far end of his apple orchard. But country wisdom notwithstanding, the town engineer, and the town bred medical officer of health, are quite of a different opinion. And so it happens that now the country is beginning to be supplied with an increased abundance of pure and disease-free water.

A very important paper was read on this subject before the Society of Engineers a few days ago by Mr. Percy Griffith, Assoc.M.Inst.C.E. Mr. Griffith took an entirely practical view of the question. In his experience he had found out that if the countryman had despised the town-bred engineer and his methods, the engineer in his turn had not, in the past, thought it worth while to devote much attention to the water supply of the few hundreds or scores of individuals who spend their lives in the hamlets, villages, and outlying market towns of the country. All that, however, is changing now; and modern engineers, finding themselves, like doctors, a great deal too thick on the ground, are glad to discover for themselves a little modestly remunerative occupation in the direction of country water supply. Mr. Griffith's paper is highly technical, and in some respects more suitable for discussion in an engineering than in a medical newspaper. But though the doctor, even if he happens not to be a medical officer of health, may not care to go very deeply into the questions of pumps, gas and water engines, suction valves, boreholes, cast-iron tanks, and the like, he is nevertheless very conscious that the actual circumstance of the water supply itself of his district is the chief of all the factors which bear upon the health of his little population. If we are permitted to summarize Mr. Griffith's paper for the doctor, it consists in the affirmations that, on the whole, deep wells are the best sources of supply for villages and small towns; and that, after the discovery of a sufficient source, the three other main questions for consideration are, the cheapest and best methods of raising the water to the surface, of distributing it to the people, and of storing the overplus, not against a "rainy" but against a "fine" day. The doctor, as everybody knows, is expected to be a man of "all-round" resources. In a very marked degree is this the case with the rural medical officer of health. We cannot help feeling that the kind of information and the illustrative examples gathered and set forth with much discriminating industry by Mr. Percy Griffith will be of great use to many of our country brethren. Moreover, it is very doubtful whether exactly the same kind of information can anywhere else be found in print, at any rate in so practical and condensed a form.

